# Protective Effects of an Oligo-Fucoidan-Based Formula against Osteoarthritis Development via iNOS and COX-2 Suppression following Monosodium Iodoacetate Injection

**DOI:** 10.3390/md22050211

**Published:** 2024-05-06

**Authors:** Yi-Fen Chiang, Ko-Chieh Huang, Kai-Lee Wang, Yun-Ju Huang, Hsin-Yuan Chen, Mohamed Ali, Tzong-Ming Shieh, Shih-Min Hsia

**Affiliations:** 1 School of Nutrition and Health Sciences, College of Nutrition, Taipei Medical University, Taipei 110301, Taiwan; 2 Department of Nursing, Deh Yu College of Nursing and Health, Keelung 203301, Taiwan; 3 Department of Biotechnology and Food Technology, Southern Taiwan University of Science and Technology, Tainan 710301, Taiwan; 4 Department of Obstetrics and Gynecology, University of Chicago, Chicago, IL 60637, USA; mohamed.ali@bsd.uchicago.edu; 5 Clinical Pharmacy Department, Faculty of Pharmacy, Ain Shams University, Cairo 11566, Egypt; 6 School of Dentistry, China Medical University, Taichung 404328, Taiwan; 7 Graduate Institute of Metabolism and Obesity Sciences, College of Nutrition, Taipei Medical University, Taipei 11031, Taiwan; 8 School of Food and Safety, Taipei Medical University, Taipei 11031, Taiwan; 9 Nutrition Research Center, Taipei Medical University Hospital, Taipei 11031, Taiwan; 10 TMU Research Center for Digestive Medicine, Taipei Medical University, Taipei 11031, Taiwan

**Keywords:** osteoarthritis, oligo-fucoidan-based formula, oxidative stress, inflammatory

## Abstract

Osteoarthritis (OA) is a debilitating joint disorder characterized by cartilage degradation and chronic inflammation, accompanied by high oxidative stress. In this study, we utilized the monosodium iodoacetate (MIA)-induced OA model to investigate the efficacy of oligo-fucoidan-based formula (FF) intervention in mitigating OA progression. Through its capacity to alleviate joint bearing function and inflammation, improvements in cartilage integrity following oligo-fucoidan-based formula intervention were observed, highlighting its protective effects against cartilage degeneration and structural damage. Furthermore, the oligo-fucoidan-based formula modulated the p38 signaling pathway, along with downregulating cyclooxygenase-2 (COX-2) and inducible nitric oxide synthase (iNOS) expression, contributing to its beneficial effects. Our study provides valuable insights into targeted interventions for OA management and calls for further clinical investigations to validate these preclinical findings and to explore the translational potential of an oligo-fucoidan-based formula in human OA patients.

## 1. Introduction

Osteoarthritis (OA), also known interchangeably as degenerative joint disease or osteoarthrosis, represents the predominant form of joint pathology. Its primary manifestations include joint pain, stiffness, swelling, and structural malformation. Additionally, abnormal joint sounds, notably crepitus, may accompany movements. In the early stages of the disease, individuals typically experience pain during routine activities such as squatting, kneeling, ascending stairs, or transitioning from seated to standing positions. As the condition progresses, this discomfort may persist during both walking and periods of rest. Moreover, prolonged immobilization in fixed postures, such as extended periods of sitting or standing, or even during nocturnal rest, may contribute to knee discomfort [1,2].

The pathogenesis of osteoarthritis (OA) involves the loss of proteoglycans and type II collagen within the articular cartilage, alongside the degradation of the extracellular matrix (ECM) [3]. This degradation leads to the deterioration of the articular cartilage, resulting in increased friction between bones, inflammation, and severe pain. Consequently, joint mobility becomes compromised, potentially leading to joint disability [4]. Accumulating evidence has demonstrated that secreted inflammatory cytokines play a central role as mediators of pathology in the progression of OA [5,6]. Interleukin-1 beta (IL-1β), tumor necrosis factor-alpha (TNF-α), and IL-6 are major factors involved in and associated with cartilage degradation. These cytokines perpetuate an inflammatory cascade, contributing to the destruction of cartilage and perpetuation of the disease process [7].

The modulation of inflammatory cytokines may occur via inducible nitric oxide synthase (iNOS) and cyclooxygenase-2 (COX-2), which can exacerbate the progression of OA by impairing cartilage function, triggering apoptosis, and worsening the disease [8].

Currently, the treatment for osteoarthritis (OA) primarily relies on medication to alleviate pain. Nonsteroidal anti-inflammatory drugs (NSAIDs) such as acetaminophen, celecoxib, and aspirin are commonly used to reduce pain and inflammation [9]. However, studies have shown that the prolonged use of NSAIDs may inhibit proteoglycan synthesis, hastening the deterioration of OA [10]. Therefore, there is a need to identify effective compounds capable of delaying proteoglycan degradation, preventing extracellular matrix (ECM) loss, and alleviating inflammatory responses.

Exploring alternative therapeutic approaches that target specific pathways involved in OA pathogenesis, such as iNOS and COX-2, could offer promising avenues for the development of more targeted and efficacious treatments. Additionally, research into natural compounds with anti-inflammatory and chondroprotective properties may provide novel therapeutic options with fewer adverse effects compared with traditional NSAIDs. Ultimately, a multifaceted approach that addresses both pain relief and disease modification is essential in effectively managing OA and improving the quality of life for affected individuals.

Previous studies have revealed the anti-inflammatory properties of low-molecular-weight fucoidan derived from brown algae extracts [11]. Fucoidan, a sulfated polysaccharide found in various species of brown seaweed, has garnered attention for its diverse biological activities, including anti-inflammatory, antioxidative, and immunomodulatory properties [12,13]. These characteristics make it an attractive candidate for exploring its efficacy in OA treatment. However, despite numerous studies highlighting its beneficial effects, the specific mechanisms by which fucoidan exerts its actions in the context of OA are not yet fully characterized. By establishing an oligo-fucoidan-based formula and determining its potential in relieving OA symptoms, we are evaluating its efficacy in mitigating OA-related pathology. By elucidating the molecular pathways through which the oligo-fucoidan-based formula exerts its protective effects on OA-affected knee joints, this study aims to provide valuable insights into its therapeutic mechanisms. These findings have the potential to inform the development of novel fucoidan-based therapies for OA management, offering new hope for patients suffering from this debilitating condition. Ultimately, a deeper understanding of the therapeutic properties of fucoidan may pave the way for personalized approaches to OA treatment that are tailored to individual patient needs.

## 2. Results

### 2.1. Formula Selection thorugh Examination of iNOS Expression (In Vitro Study)

**Figure 1:**
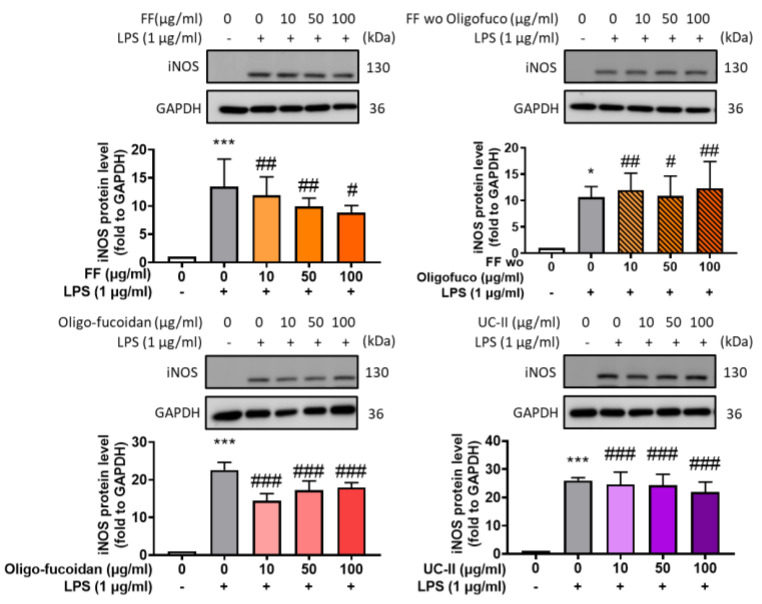
iNOS expression modulation by FF and FF-related components. Raw 264.7 cells were treated with the oligo-fucoidan-based formula (FF) and its related components and then induced by LPS for 24 h. Western blot was used to evaluate the iNOS expression. * *p* < 0.05; *** *p* < 0.001 compared with the control group. # *p* < 0.05; ## *p* < 0.01; ### *p* < 0.001 compared with the LPS-induced group.

To determine whether the formula has a greater effect than its individual components, we measured the iNOS expression in response to the oligo-fucoidan-based formula (FF) and its individual components, including the formula without oligo-fucoidan, oligo-fucoidan, and UC-II. Experimenting with Raw264.7 cells induced with LPS, we observed that the FF displayed superior inhibition of iNOS production compared with the individual fucoidan and UC-II components, which exhibited only partial inhibition abilities (Figure 1).

### 2.2. Protective Effect of the Oligo-Fucoidan-Based Formula (FF) on Joint Swelling Induced by MIA-Induced Osteoarthritis

**Figure 2:**
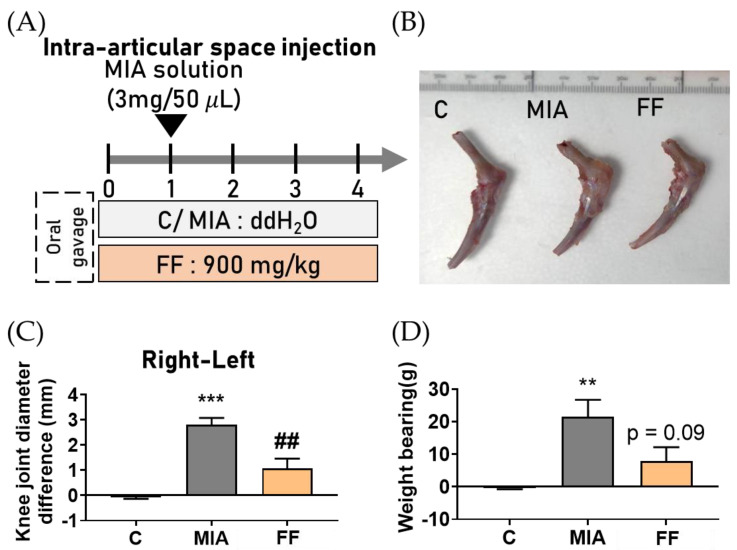
The effects of oligo-fucoidan-based formula intervention on the symptoms of OA. (**A**) Flow chart of the procedure. (**B**) Knee appearance after treatment. (**C**) Knee diameter difference. (**D**) Weight bearing ability. The experimental groups were the following: C, control group; MIA, MIA-induced group; FF, oligo-fucoidan-based formula (FF) 900 mg/kg + MIA-induced group. Statistical significance is denoted as ** *p* < 0.01; *** *p* < 0.001 compared with the control group, and ## *p* < 0.01 compared with the MIA-induced group.

The flow chart of procedures was shown in Figure 2A. Three weeks after intervention with the oligo-fucoidan-based formula, the width of the knee joint in the hind limbs was measured using a caliper. The results demonstrated that intervention with the oligo-fucoidan-based formula effectively reduced the occurrence of joint swelling induced by MIA (Figure 2B,C).

The data obtained from the incapacitance tester were calculated using the following formula: the pressure ratio between the non-induced side and the MIA-induced side was determined to assess the difference in hind-limb pressure [14]. The results indicated a significant transfer of pressure to the non-induced side in the MIA-induced group, while the groups treated with the oligo-fucoidan-based formula exhibited a trend t restoring joint function (Figure 2D). These results indicated that the oligo-fucoidan-based formula has a potential protective effect on MIA-induced osteoarthritis symptoms.

### 2.3. Oligo-Fucoidan-Based Formula Toxicity Evaluation

**Figure 3:**
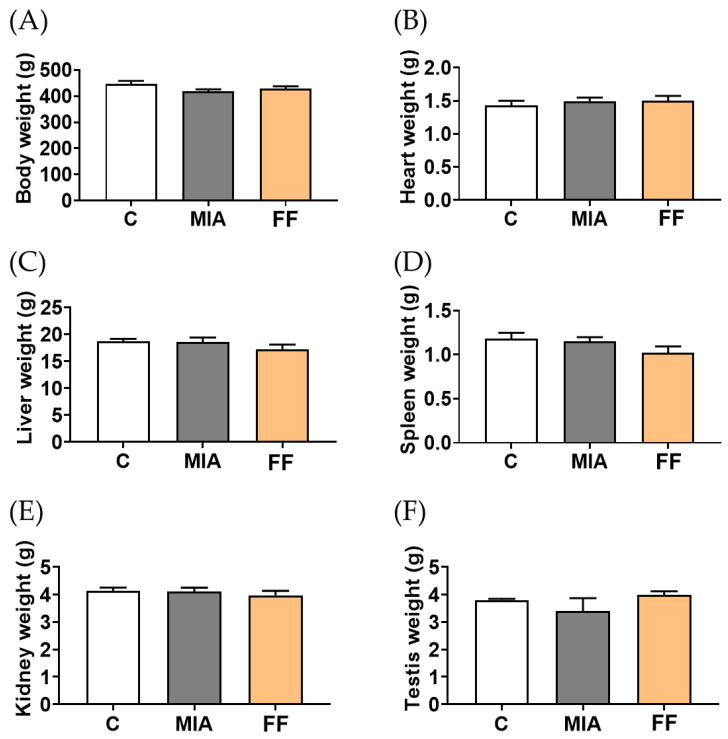
The toxicity evaluation of oligo-fucoidan-based formula intervention. The examination of body weight (**A**), heart (**B**), liver (**C**), spleen (**D**), kidney (**E**), and testis weight (**F**) showed no significant differences after the intervention. The experimental groups were the following: C, control group; MIA, MIA-induced group; FF, oligo-fucoidan-based formula (FF) 900 mg/kg + MIA-induced group.

Changes in organ weight are recognized as highly sensitive indicators of organ damage in determining compound toxicity [15]. The results revealed that following 4 weeks of treatment, the administration of the oligo-fucoidan-based formula had no distinct effect on body weight or on the weight changes of other major organs, including the heart, liver, spleen, kidneys, and testes. These findings suggest that prolonged intervention with the oligo-fucoidan-based formula would not cause any toxicity (Figure 3).

### 2.4. Cytokine Secretion and Malondialdehyde (MDA) Concentration

**Figure 4:**
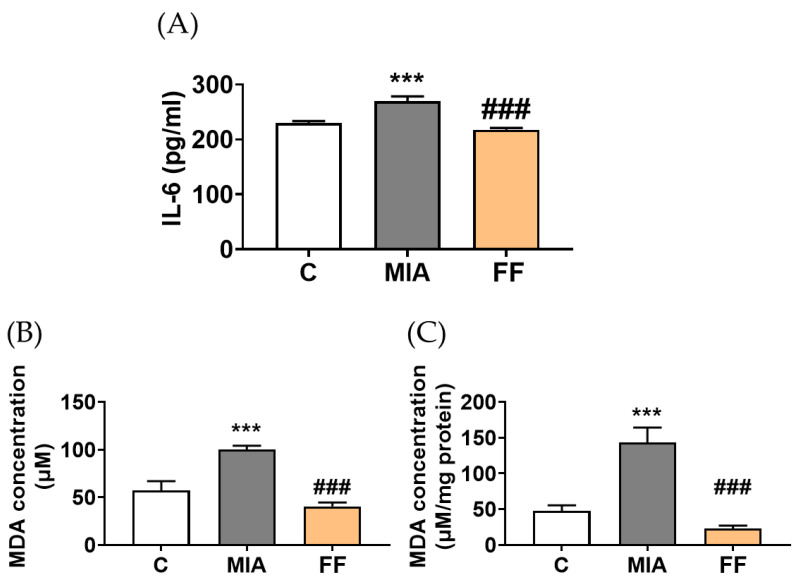
Cytokine secretion and oxidative stress evaluation. (**A**) IL-6 levels were evaluated after the intervention. (**B**) The serum MDA concentration and (**C**) tissue MDA concentration were evaluated by the TBARS assay. The experimental groups were the following: C, control group; MIA, MIA-induced group; FF, oligo-fucoidan-based formula (FF) 900 mg/kg + MIA-induced group. Statistical significance is denoted as *** indicating *p* < 0.001 compared with the control group, and ### indicating *p* < 0.001 compared with the MIA-induced group.

Due to the inflammatory cytokines induced by MIA playing a critical role as mediators of pathology in the progression of OA, it was essential to explore whether the oligo-fucoidan-based formula could alleviate cytokine secretion. The serum level of the inflammatory cytokine IL-6 was analyzed for this purpose. The results showed that MIA-induced osteoarthritis significantly increased the IL-6 content, enhancing its inflammatory response. However, after three weeks of intervention with the oligo-fucoidan-based formula, the inflammatory response induced by MIA was significantly restored, indicating its potential to delay joint pain and swelling, possibly through reducing inflammation (Figure 4A).

Elevated levels of IL-6 may induce the production of reactive oxygen species (ROS), leading to the degradation of the cartilage extracellular matrix and subsequent joint dysfunction [16]. To understand the effect of the oligo-fucoidan-based formula on ROS release, the measurement of malondialdehyde (MDA) was conducted, revealing a potential oxygen radical activity that is indicative of an inflammatory status [17]. As shown in Figure 4B,C, the generation of MDA was observed in the MIA group both in serum and tissue, while oligo-fucoidan-based formula intervention successfully decreased the MDA concentration. The results suggest that the oligo-fucoidan-based formula had a preventive ability on MIA-induced osteoarthritis via the inhibition of inflammation and oxidative stress.

### 2.5. Histological Assessment

**Figure 5:**
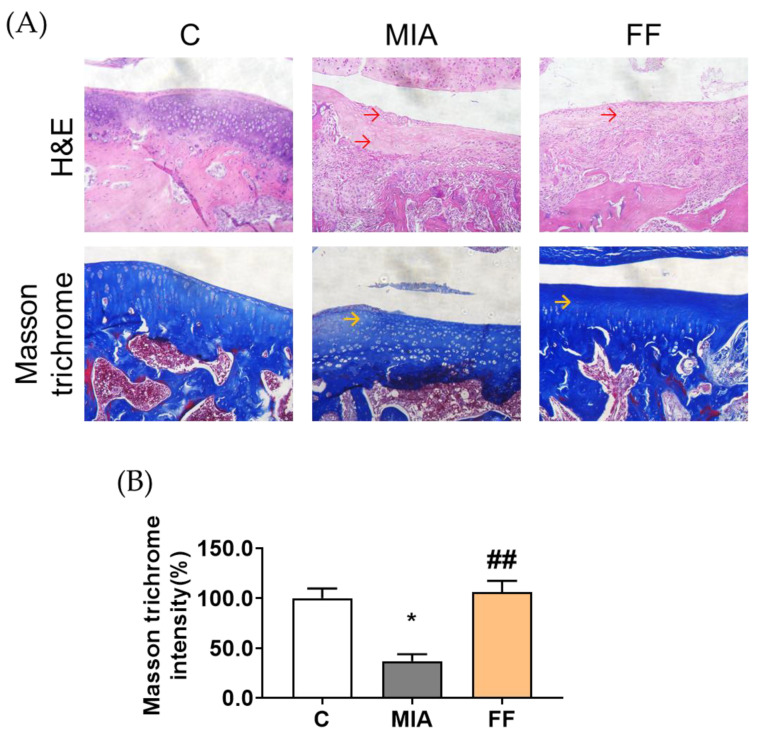
Histological appearance of the articular cartilage after the intervention. (**A**) Histological image after staining with H&E and Masson trichrome. (**B**) Quantification of the Masson trichrome intensity. The experimental groups were the following: C, control group; MIA, MIA-induced group; FF, oligo-fucoidan-based formula (FF) 900 mg/kg + MIA-induced group. Red arrow: articular cartilage showing some irregularities on the surface. Yellow arrow: extracellular matrix leakage. Statistical significance is denoted as * indicating *p* < 0.05 compared with the control group, and ##, indicating *p* < 0.01 compared with the MIA-induced group.

Next, in order to understand the histology of cartilage integrity and to explore whether the oligo-fucoidan-based formula could have a recovery effect on MIA-induced osteoarthritis, we utilized histological assessment by employing hematoxylin and eosin (H&E) staining and Masson’s trichrome staining in the articular cartilage. In the MIA group, the articular cartilage displayed surface irregularities (as shown by the red arrow), accompanied by extracellular matrix leakage (as shown by the yellow arrow), compared with the control group (Figure 5). These observations suggest the initiation of cartilage degeneration and structural damage caused by MIA.

In contrast, there was an opposite consequence in the oligo-fucoidan-based formula group; the articular cartilage appeared more preserved, with fewer surface irregularities and reduced extracellular matrix leakage as shown in the quantitative results (Figure 5). This observation suggests that oligo-fucoidan-based formula intervention may help mitigate cartilage degeneration and maintain structural integrity, potentially contributing to improved joint health.

### 2.6. Modulation of Related Pathways

To comprehensively examine the potential modulation effects and molecular pathways influenced by the oligo-fucoidan-based formula in MIA-induced osteoarthritis, the protein expression of key markers, including phosphorylated p38 (p-p38), inducible nitric oxide synthase (iNOS), and cyclooxygenase-2 (COX-2), were assessed using Western blot analysis.

**Figure 6:**
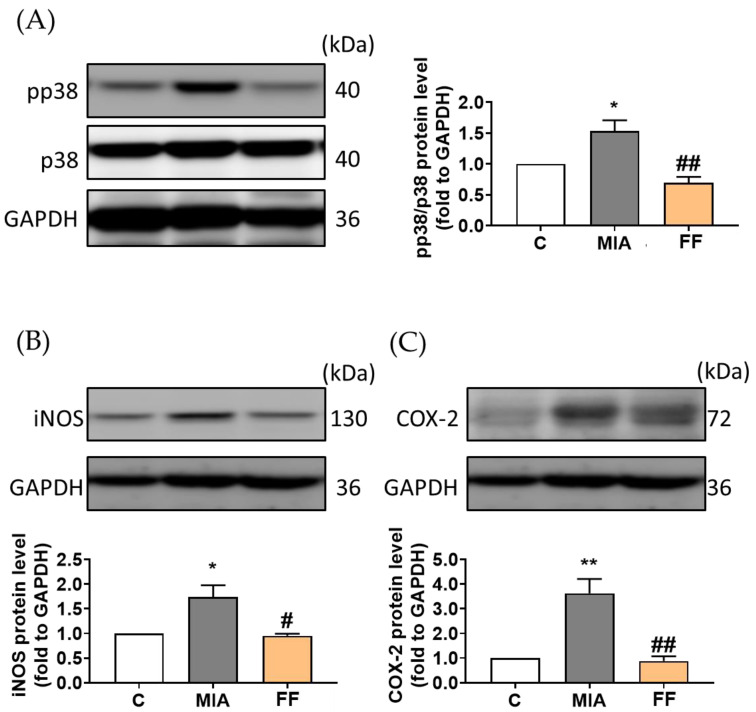
Related pathway modulation in oligo-fucoidan-based formula modulation. (**A**) pp38/p38, (**B**) iNOS, and (**C**) COX-2 protein expression was examined by Western blotting. The experimental groups were the following: C, control group; MIA, MIA-induced group; FF, oligo-fucoidan-based formula (FF) 900 mg/kg + MIA-induced group. Statistical significance is denoted as * *p* <0.05; ** *p* < 0.01 compared with the control group, and # *p* < 0.05; ## *p* <0.01 compared with the MIA-induced group.

The results revealed that in the MIA model group, there was a notable increase in the protein expression of phosphorylated p38, iNOS, and COX-2, while oligo-fucoidan-based formula intervention demonstrated a significant reduction in p-p38 signaling, along with decreased protein expression of downstream targets such as iNOS and COX-2. These findings suggest that the oligo-fucoidan-based formula may exert its effects by modulating these pathways, thereby potentially contributing to its anti-inflammatory and protective properties in the context of joint health (Figure 6).

## 3. Discussion

Here, we present evidence supporting the protective role of an oligo-fucoidan-based formula in the progression of osteoarthritis (OA). The oligo-fucoidan-based formula acts by modulating the p38 signaling pathway and reducing the expression of COX-2 and iNOS, which enables it to alleviate joint burden and inflammation. This indicates its potential to mitigate OA progression by targeting key inflammatory pathways and processes involved in joint degeneration.

Excessive oxidative stress production may occur during the progression of OA due to the relatively low oxygen supply in cartilage [18]. This imbalance between the production of reactive oxygen species (ROS) and antioxidative defense mechanisms in the joint tissue can lead to oxidative damage and contribute to the pathogenesis of OA. In the context of OA pathology, situations such as ischemia–reperfusion phenomena can trigger oxygen responses, further exacerbating ROS production. ROS, including superoxide anions, hydrogen peroxide, and hydroxyl radicals, are highly reactive molecules that can cause damage to cellular components [19]. This oxidative damage can disrupt the normal functioning of chondrocytes, leading to cartilage degradation and joint dysfunction. Additionally, ROS can stimulate inflammatory responses by activating signaling pathways involved in the production of pro-inflammatory cytokines and mediators [20]. Thus, the interplay between oxidative stress and inflammation accelerates and participates in the crucial pathogenesis of OA, highlighting the importance of targeting ROS-mediated pathways for therapeutic intervention in OA management.

Clinical treatments for OA are divided into medication and dietary supplementation. Medications commonly prescribed for OA management include the non-steroidal anti-inflammatory drugs (NSAIDs), acetaminophen, and serotonin–norepinephrine reuptake inhibitors (SNRIs). However, it is crucial to acknowledge the potential side effects associated with these medications [21]. Acetaminophen, while effective in managing pain, carries the risk of causing liver damage, elevating transient liver enzymes, and inducing hepatotoxicity [22]. NSAIDs, often used to alleviate pain and inflammation, can lead to gastrointestinal discomfort and may exacerbate pre-existing kidney conditions [23]. SNRIs, which are primarily indicated for treating depression and mood disorders, can result in adverse effects such as fatigue and sexual dysfunction [24].

These side effects underscore the need for alternative treatment options with fewer adverse reactions. Natural compounds with antioxidative properties have shown advantages in alleviating OA progression through animal studies. Resveratrol, a natural polyphenol compound, has demonstrated the ability to restore chondrocyte apoptosis, alleviate oxidative stress, and improve disease progression [25,26]. Galangin, a bioactive flavonoid, has been shown to minimize ROS production, elevate antioxidative enzyme levels, and reduce inflammatory cytokines related to OA. These effects contribute to an improvement in OA performance [27]. By harnessing the therapeutic potential of nature’s resources, we can provide patients with OA relief while minimizing the risk of adverse reactions, ultimately improving their quality of life.

Fucoidan, a prominent component of the oligo-fucoidan-based formula, has garnered attention for its multifaceted biological properties. Studies have revealed its ability to regulate key signaling pathways such as the MAPK pathway, which plays a pivotal role in mediating cellular responses to various stimuli, including inflammation and oxidative stress. The MAPK pathway encompasses a cascade of protein kinase reactions that ultimately regulate gene expression, cell proliferation, differentiation, and apoptosis. By modulating the MAPK signaling transduction, fucoidan can exert antioxidative effects, thereby mitigating tissue damage and promoting cellular resilience. Moreover, fucoidan has been demonstrated to attenuate ROS generation, further enhancing its protective capabilities against oxidative damage. While ROS play essential roles in cellular signaling and host defense mechanisms, excessive ROS production can lead to oxidative stress, resulting in damage to lipids, proteins, and DNA. In the context of osteoarthritis (OA), oxidative stress contributes to cartilage degradation, inflammation, and joint tissue damage [28]. Fucoidan’s ability to scavenge ROS and modulate antioxidative enzyme activity helps maintain the redox balance within the joint microenvironment, thereby alleviating OA symptoms and slowing disease progression. These properties of fucoidan extend beyond joint health, as evidenced by its efficacy in protecting against retinal harm. Oxidative stress in the retina contributes to the development and progression of various eye disease, including age-related macular degeneration (AMD), diabetic retinopathy, and glaucoma [29]. Fucoidan’s antioxidative properties help mitigate retinal oxidative damage, thereby preserving retinal function and preventing the vision loss associated with these conditions [30].

In OA, the expression of inducible nitric oxide synthase (iNOS) and cyclooxygenase-2 (COX-2) is regulated by the MAPK signaling pathway, which is modulated by cytokines and the breakdown of the extracellular matrix. In our study, we observed that oligo-fucoidan-based formula intervention suppressed inflammation and downregulated the expression of phosphorylated p38 (p-p38), iNOS, and COX-2 [31]. These findings suggest a modulatory role for the oligo-fucoidan-based formula in attenuating OA-associated inflammation and ECM degradation through the regulation of the MAPK signaling pathway.

Moreover, pain associated with OA could be exacerbated by the presence of ROS, which activate MAPK signaling and modulate COX-2 expression. MIA, acting as an inhibitor of glyceraldehyde-3-phosphate dehydrogenase, induces cartilage degradation, leading to ROS accumulation and inflammation. This pathological mechanism closely resembles the progression of OA, where cartilage degradation, ROS accumulation, and inflammation contribute to the development and exacerbation of pain symptoms [32].

With the observation of the downregulation of p-p38, inducible nitric oxide synthase (iNOS), and COX-2 expression after oligo-fucoidan-based formula treatment, potential improvement by the oligo-fucoidan-based formula in the monosodium iodoacetate (MIA)-induced animal model was suggested. This was accompanied by modulation of the MAPK pathway and the regulation of inflammatory cytokine release. These findings demonstrate the potential role of the oligo-fucoidan-based formula as a therapeutic agent in OA management.

Previous studies have demonstrated the multifaceted modulatory capabilities of fucoidan, including its formulation’s role in reducing COX-2 expression and cytokine secretion [33]. Through its modulation of iNOS expression and IL-6 secretion and its reduction of MAPK signaling, fucoidan exhibits promising potential in relieving symptoms associated with OA [34]. These findings underscore the broad therapeutic potential of fucoidan in addressing the complex pathophysiology of OA and highlight its role as a promising candidate for the development of OA-relieving interventions.

The remaining 70% of the oligo-fucoidan-based formula consists of UC-II^®^, undenatured type II collagen, hyaluronic acid, chondroitin, calcium carbonate, glucosamine, and excipients. These components have been reported to have potential effectiveness in OA progression. Among them, UC-II has been found to be the most effective and is one of the most commonly used supplements on the market [35]. Additionally, a study demonstrated inflammatory alleviation and pain relief by UC-II in combination with glucosamine hydrochloride and chondroitin sulfate during a 150-day treatment period [36]. However, due to commercial confidentiality and the limitations in these studies, we cannot conclusively determine the effectiveness and synergistic effect of UC-II or of oligo-fucoidan. Nevertheless, it is worth noting that the oligo-fucoidan-based formula may offer enhanced antioxidative and anti-inflammatory abilities, potentially contributing to improved OA progression. Nevertheless, further studies are warranted to fully elucidate its efficacy and potential benefits in clinical settings.

This study highlights the potential functional efficacy of the oligo-fucoidan-based formula for osteoarthritis, underscoring its role as a promising substance. Given the multifactorial nature of OA, which involves many mechanisms, there is a growing interest in exploring novel treatment approaches using advanced” omics” tools, including genomics, proteomics, metabolomics, and transcriptomics. Building upon these findings, further studies employing multi-omics technologies will be crucial in investigating the underlying mechanisms of action of the oligo-fucoidan-based formula in OA treatment, thereby offering valuable insights into its therapeutic potential [37,38].

## 4. Materials and Methods

### 4.1. Reagents

The oligo-fucoidan-based formula (FF) provided by HiQ Marine Biotech (Taipei, Taiwan) and branded as Joint-free^®^, comprises UC-II^®^ and undenatured type II collagen, along with food-grade effective hyaluronic acid, high-purity chondroitin, oligo-fucoidan (OliFuco^®^), calcium carbonate, and glucosamine. Its key ingredient is oligo-fucoidan (comprising more than 30% of the formula), derived from naturally dried oceanic brown seaweed; specifically, Laminaria japonica.

In brief, the preparation of oligo-fucoidan involves subjecting the crude extract of Laminaria japonica to elution with a sodium chloride gradient using a DEAE (diethylaminoethyl)-Sephadex A-25 column. The fucose- and sulfate-enriched fraction is then preserved by hydrolyzing it with glycolytic enzymes [39] to an average MW of 1.2 kDa (~90.1%) [40]. The components of oligo-fucoidan have been reported to consist of sulfate and neutral monosaccharides comprising fucose, glucose, galactose, myo-inositol, mannose, xylose, and rhamnose [40].

### 4.2. Cell Culture and Treatment

Raw264.7 cells were kindly provided by Professor Yong-Han Hong (National Taiwan Normal University, Taipei, Taiwan). These were cultured in DMEM(HG) (Gibco, Life Technologies, Carlsbad, CA, USA) supplemented with 10% fetal bovine serum (FBS) (Corning, New York, NY, USA) and a 1% Antibiotic-Antimycotic (Corning) solution in a 5% CO_2_, 37 °C incubator. The aim was to evaluate the formula and the related components’ ability in suppressing iNOS expression. The FF, FF without oligo-fucoidan, oligo-fucoidan, and UC-II supplied from Hi-Q were co-treated with 1 μg/mL of lipopolysaccharides from Escherichia coli O111:B4 (Sigma Aldrich, St. Louis, MO, USA), based on a previous study [41].

### 4.3. Animal Osteoarthritis Model and Evaluation

Four-week-old Wistar rats were used in this study. Osteoarthritis was induced by the intra-articular injection of 3 mg of monosodium iodoacetate (MIA, Sigma-Aldrich, St. Louis, MO, USA) dissolved in 50 μL of 0.9% saline into the ligament of the right knees using a 29-gauge, 0.5-inch needle, while the control group received saline injections [42,43]. Body weight and knee joint width were measured prior to injection to establish baseline values. All animal procedures were approved by the IAUAC (No: LAC-2021-0308). The experimental group received the oligo-fucoidan-based formula (FF group), which was administered orally via oral gavage (0.9 mL/rat dissolved in ddH_2_O) for 4 weeks, beginning one week before the injection and continuing starting the day after injection, while the control group received standard care. Body weight and knee joint width were monitored weekly post-injection to assess disease progression. Bilateral weight-bearing pressure distribution was evaluated every two weeks using the incapacitance test to measure pain and functional impairment.

### 4.4. Bilateral Weight-Bearing Test

The bilateral weight-bearing test is employed to quantify weight distribution during the hind-limb stance and serves as an indicator for assessing joint pain and discomfort in animals. In physiological conditions, rats typically exhibit an equitable weight distribution across their hind limbs, resulting in a bilateral pressure difference approaching zero. Conversely, unilateral knee joint injury in rats necessitates the non-injured hind limb to support a greater portion of the body weight, leading to an elevation in the bilateral pressure difference attributable to pain sensation. The Incapacitance tester (Ugo Basile Biological Instruments, Gemonio, Italy), designed specifically for this purpose, facilitates the measurement of hind-limb weight distribution. Rats undergo preliminary training to stand on their hind limbs within a force plate box outfitted with a 65° inclined plane. Subsequently, during formal testing, this force plate box is positioned atop the bilateral weight-bearing test apparatus, enabling rats to autonomously assume a hind-limb stance. The bilateral weight-bearing test apparatus then records and analyzes the pressure difference between the two hind limbs individually. Following five repetitions of this procedure, the average pressure difference is computed to derive a representative value.

### 4.5. Measurement of Knee Joint Width

Knee joint width was measured from both right and left knees using an electronic digital caliper.

### 4.6. IL-6 Level Measurement

The evaluation of serum levels of the inflammatory cytokine IL-6 was performed using a commercially available enzyme-linked immunosorbent assay (ELISA) kit obtained from Abcam (Cambridge, UK). The procedure was conducted following the manufacturer’s instructions.

### 4.7. Thiobarbituric Acid-Reactive Substances (TBARS) Assay

The assessment of malondialdehyde (MDA) concentration was conducted using a commercially available assay kit obtained from Cayman Chemical (Ann Arbor, MI, USA). The procedure was carried out according to the manufacturer’s instructions. Briefly, samples were treated with sodium dodecyl sulfate (SDS), followed by the addition of sodium hydroxide (NaOH) and 2-thiobarbituric acid (TBA). The mixture was then subjected to boiling at 95 °C for 1 h. Absorbance measurements were performed at the 532 nm wavelength.

### 4.8. Histological Examination

Following sacrifice, knee joints were harvested and fixed in 10% formalin solution for 48 h. Subsequently, the samples underwent dehydration with ethanol, embedding in paraffin, and sectioning into 4 μm slices for staining. Hematoxylin and eosin (H&E), as well as Masson’s trichrome staining procedures, were conducted by Bio-Check Laboratories Ltd. (Taipei, Taiwan). Masson’s trichrome staining marks collagen via blue staining, and Image J was used for quantifying the blue area [44].

### 4.9. Western Blot

The hind-limb knee joints were excised and lysed using lysis buffer containing RIPA supplemented with phosphatase and protease inhibitors (Roche, Basel, Switzerland). The tissue was homogenized using a homogenizer. Protein quantification was performed using the BCA assay (T-Pro Biotechnology, New Taipei City, Taiwan). Subsequently, sodium dodecyl sulfate polyacrylamide gel electrophoresis (SDS-PAGE) was utilized for protein separation, followed by transfer to a polyvinylidene fluoride (PVDF) membrane (Millipore Sigma, Billerica, MA, USA) using a Bio-Rad equivalent system (Bio-Rad, Hercules, USA). Blocking was carried out using 5% BSA (Biomax, Taipei, Taiwan). The primary antibodies against pp38 and p38 from Cell Signaling (Boston, MA, USA) and against iNOS, COX-2, and GAPDH from Proteintech (Rosemont, IL, USA) were incubated overnight at 4 °C. After washing thrice for 10 min each with TBST (T-Pro), an HRP-conjugated goat anti-rabbit IgG secondary antibody (Jackson ImmunoResearch Laboratories, West Grove, PA, USA) was applied for 2 h at room temperature. Signal capture was performed using the eBlot Touch Imager™ (eBlot Photoelectric Technology, Shanghai, China), and quantification was conducted using Image J software (Version 1.53t, NIH, Bethesda, MD, USA).

### 4.10. Statistical Analysis

The data are presented as the mean ± standard error of the mean (SEM). Statistical analyses were performed using GraphPad Prism, version 9.0. The student’s *t*-test and one-way analysis of variance (ANOVA) followed by Tukey’s post hoc test were employed for comparisons. Results with a *p*-value of less than 0.05 were considered statistically significant.

## 5. Conclusions

In conclusion, our study underscores the efficacy of oligo-fucoidan-based formula (FF) intervention in mitigating the progression of OA. Through its ability to alleviate joint burden and inflammation, the oligo-fucoidan-based formula shows promise as a therapeutic intervention. Histological assessments revealed substantial improvements in cartilage integrity following oligo-fucoidan-based formula intervention, indicating its protective effects against cartilage degeneration and structural damage. Furthermore, the modulation of the p38 signaling pathway, coupled with the downregulation of COX-2 and iNOS expression, sheds light on the mechanistic insights underlying oligo-fucoidan-based formula’s beneficial effects. These findings underscore the therapeutic potential of the oligo-fucoidan-based formula in mitigating OA-associated joint dysfunction and inflammation, offering new avenues for targeted interventions in OA management. Further clinical investigations are warranted to validate these preclinical findings and to explore the translational potential of the oligo-fucoidan-based formula in OA patients.

## Data Availability

The data presented in this study are available on request from the corresponding author.

## References

[1] Tonge D.P., Pearson M.J., Jones S.W. (2014). The hallmarks of osteoarthritis and the potential to develop personalised disease-modifying pharmacological therapeutics. Osteoarthr. Cartil..

[2] Philp A.M., Davis E.T., Jones S.W. (2017). Developing anti-inflammatory therapeutics for patients with osteoarthritis. Rheumatology.

[3] Tekari A., Luginbuehl R., Hofstetter W., Egli R.J. (2014). Chondrocytes expressing intracellular collagen type ii enter the cell cycle and co-express collagen type i in monolayer culture. J. Orthop. Res..

[4] Cicuttini F.M., Wluka A.E. (2014). Osteoarthritis: Is oa a mechanical or systemic disease?. Nat. Rev. Rheumatol..

[5] Molnar V., Matišić V., Kodvanj I., Bjelica R., Jeleč Ž., Hudetz D., Rod E., Čukelj F., Vrdoljak T., Vidović D. (2021). Cytokines and chemokines involved in osteoarthritis pathogenesis. Int. J. Mol. Sci..

[6] Kapoor M., Martel-Pelletier J., Lajeunesse D., Pelletier J.P., Fahmi H. (2011). Role of proinflammatory cytokines in the pathophysiology of osteoarthritis. Nat. Rev. Rheumatol..

[7] Shin M.R., Lee J.A., Kim M.J., Park H.J., Park B.W., Seo S.B., Roh S.S. (2020). Protective effects of *Phellinus linteus* mycelium on the development of osteoarthritis after monosodium iodoacetate injection. Evid. Based Complement. Alternat. Med..

[8] Lee Y.T., Yunus M.H.M., Ugusman A., Yazid M.D. (2022). Natural compounds affecting inflammatory pathways of osteoarthritis. Antioxidants.

[9] Bijlsma J.W., Berenbaum F., Lafeber F.P. (2011). Osteoarthritis: An update with relevance for clinical practice. Lancet.

[10] Brandt K.D. (1987). Effects of nonsteroidal anti-inflammatory drugs on chondrocyte metabolism in vitro and in vivo. Am. J. Med..

[11] Park H.Y., Han M.H., Park C., Jin C.Y., Kim G.Y., Choi I.W., Kim N.D., Nam T.J., Kwon T.K., Choi Y.H. (2011). Anti-inflammatory effects of fucoidan through inhibition of nf-κb, mapk and akt activation in lipopolysaccharide-induced bv2 microglia cells. Food Chem. Toxicol. Int. J. Publ. Br. Ind. Biol. Res. Assoc..

[12] Chau Y.T., Chen H.Y., Lin P.H., Hsia S.M. (2019). Preventive effects of fucoidan and fucoxanthin on hyperuricemic rats induced by potassium oxonate. Mar. Drugs.

[13] Vasarri M., Barletta E., Degl’Innocenti D. (2022). Marine migrastatics: A comprehensive 2022 update. Mar. Drugs.

[14] Di Cesare Mannelli L., Micheli L., Zanardelli M., Ghelardini C. (2013). Low dose native type ii collagen prevents pain in a rat osteoarthritis model. BMC Musculoskelet. Disord..

[15] Lazic S.E., Semenova E., Williams D.P. (2020). Determining organ weight toxicity with bayesian causal models: Improving on the analysis of relative organ weights. Sci. Rep..

[16] Ansari M.Y., Ahmad N., Haqqi T.M. (2020). Oxidative stress and inflammation in osteoarthritis pathogenesis: Role of polyphenols. Biomed. Pharmacother..

[17] Cherian D.A., Peter T., Narayanan A., Madhavan S.S., Achammada S., Vynat G.P. (2019). Malondialdehyde as a marker of oxidative stress in periodontitis patients. J. Pharm. Bioallied Sci..

[18] Blake D.R., Merry P., Unsworth J., Kidd B.L., Outhwaite J.M., Ballard R., Morris C.J., Gray L., Lunec J. (1989). Hypoxic-reperfusion injury in the inflamed human joint. Lancet.

[19] Koike M., Nojiri H., Ozawa Y., Watanabe K., Muramatsu Y., Kaneko H., Morikawa D., Kobayashi K., Saita Y., Sasho T. (2015). Mechanical overloading causes mitochondrial superoxide and sod2 imbalance in chondrocytes resulting in cartilage degeneration. Sci. Rep..

[20] Mathy-Hartert M., Deby-Dupont G.P., Reginster J.Y., Ayache N., Pujol J.P., Henrotin Y.E. (2002). Regulation by reactive oxygen species of interleukin-1beta, nitric oxide and prostaglandin e(2) production by human chondrocytes. Osteoarthr. Cartil..

[21] Zhang W., Robertson W.B., Zhao J., Chen W., Xu J. (2019). Emerging trend in the pharmacotherapy of osteoarthritis. Front. Endocrinol..

[22] Hochberg M.C., Altman R.D., April K.T., Benkhalti M., Guyatt G., McGowan J., Towheed T., Welch V., Wells G., Tugwell P. (2012). American college of rheumatology 2012 recommendations for the use of nonpharmacologic and pharmacologic therapies in osteoarthritis of the hand, hip, and knee. Arthritis Care Res..

[23] Bally M., Dendukuri N., Rich B., Nadeau L., Helin-Salmivaara A., Garbe E., Brophy J.M. (2017). Risk of acute myocardial infarction with nsaids in real world use: Bayesian meta-analysis of individual patient data. BMJ.

[24] da Costa B.R., Nüesch E., Reichenbach S., Jüni P., Rutjes A.W. (2012). Doxycycline for osteoarthritis of the knee or hip. Cochrane Database Syst. Rev..

[25] Wang J., Gao J.S., Chen J.W., Li F., Tian J. (2012). Effect of resveratrol on cartilage protection and apoptosis inhibition in experimental osteoarthritis of rabbit. Rheumatol. Int..

[26] Wei Y., Jia J., Jin X., Tong W., Tian H. (2018). Resveratrol ameliorates inflammatory damage and protects against osteoarthritis in a rat model of osteoarthritis. Mol. Med. Rep..

[27] Su Y., Shen L., Xue J., Zou J., Wan D., Shi Z. (2021). Therapeutic evaluation of galangin on cartilage protection and analgesic activity in a rat model of osteoarthritis. Electron. J. Biotechnol..

[28] Lepetsos P., Papavassiliou A.G. (2016). Ros/oxidative stress signaling in osteoarthritis. Biochim. Biophys. Acta.

[29] Wang K., Chen Y.S., Chien H.W., Chiou H.L., Yang S.F., Hsieh Y.H. (2022). Melatonin inhibits naio(3)-induced arpe-19 cell apoptosis via suppression of hif-1α/bnip3-lc3b/mitophagy signaling. Cell Biosci..

[30] Li X., Zhao H., Wang Q., Liang H., Jiang X. (2015). Fucoidan protects arpe-19 cells from oxidative stress via normalization of reactive oxygen species generation through the ca^2+^-dependent erk signaling pathway. Mol. Med. Rep..

[31] Meng X., Sun L., Meng X., Bi Q. (2024). The protective effect of ergolide in osteoarthritis: In vitro and in vivo studies. Int. Immunopharmacol..

[32] Zahan O.M., Serban O., Gherman C., Fodor D. (2020). The evaluation of oxidative stress in osteoarthritis. Med. Pharm. Rep..

[33] Manikandan R., Parimalanandhini D., Mahalakshmi K., Beulaja M., Arumugam M., Janarthanan S., Palanisamy S., You S., Prabhu N.M. (2020). Studies on isolation, characterization of fucoidan from brown algae turbinaria decurrens and evaluation of it’s in vivo and in vitro anti-inflammatory activities. Int. J. Biol. Macromol..

[34] Apostolova E., Lukova P., Baldzhieva A., Katsarov P., Nikolova M., Iliev I., Peychev L., Trica B., Oancea F., Delattre C. (2020). Immunomodulatory and anti-inflammatory effects of fucoidan: A review. Polymers.

[35] Gencoglu H., Orhan C., Sahin E., Sahin K. (2020). Undenatured type ii collagen (uc-ii) in joint health and disease: A review on the current knowledge of companion animals. Animals.

[36] Gupta R.C., Canerdy T.D., Lindley J., Konemann M., Minniear J., Carroll B.A., Hendrick C., Goad J.T., Rohde K., Doss R. (2012). Comparative therapeutic efficacy and safety of type-ii collagen (uc-ii), glucosamine and chondroitin in arthritic dogs: Pain evaluation by ground force plate. J. Anim. Physiol. Anim. Nutr..

[37] Gonzalez-Alvarez M.E., Sanchez-Romero E.A., Turroni S., Fernandez-Carnero J., Villafañe J.H. (2023). Correlation between the altered gut microbiome and lifestyle interventions in chronic widespread pain patients: A systematic review. Medicina.

[38] Kraus V.B., Reed A., Soderblom E.J., Moseley M.A., Hsueh M.F., Attur M.G., Samuels J., Abramson S.B., Li Y.J. (2024). Serum proteomic panel validated for prediction of knee osteoarthritis progression. Osteoarthr. Cartil. Open.

[39] Liao C.H., Lai I.C., Kuo H.C., Chuang S.E., Lee H.L., Whang-Peng J., Yao C.J., Lai G.M. (2019). Epigenetic modification and differentiation induction of malignant glioma cells by oligo-fucoidan. Mar. Drugs.

[40] Chen L.M., Yang P.P., Al Haq A.T., Hwang P.A., Lai Y.C., Weng Y.S., Chen M.A., Hsu H.L. (2022). Oligo-fucoidan supplementation enhances the effect of olaparib on preventing metastasis and recurrence of triple-negative breast cancer in mice. J. Biomed. Sci..

[41] Cao Y., Chen J., Ren G., Zhang Y., Tan X., Yang L. (2019). Punicalagin prevents inflammation in lps-induced raw264.7 macrophages by inhibiting foxo3a/autophagy signaling pathway. Nutrients.

[42] Takahashi I., Matsuzaki T., Kuroki H., Hoso M. (2018). Induction of osteoarthritis by injecting monosodium iodoacetate into the patellofemoral joint of an experimental rat model. PLoS ONE.

[43] Pitcher T., Sousa-Valente J., Malcangio M. (2016). The monoiodoacetate model of osteoarthritis pain in the mouse. J. Vis. Exp..

[44] Chen Y., Yu Q., Xu C.-B. (2017). A convenient method for quantifying collagen fibers in atherosclerotic lesions by imagej software. Int. J. Clin. Exp. Med..

